# Assessment of Steel Storage Tank Thickness Obtained from the API 650 Design Procedure Through Nonlinear Dynamic Analysis, Accounting for Large Deformation Effects

**DOI:** 10.3390/ma18010066

**Published:** 2024-12-27

**Authors:** Sobhan Fallah Daryavarsari, Roberto Nascimbene

**Affiliations:** 1 Department DICAr, University of Pavia, 27100 Pavia, Italy; sobhan.fallahdaryavar01@universitadipavia.it; 2 Istituto Universitario di Studi Superiori IUSS—Department STS, Scuola Universitaria Superiore Pavia, 27100 Pavia, Italy

**Keywords:** nonlinear dynamic analysis, spectral analysis, API 650, steel storage tanks, seismic vulnerability, elephant-foot buckling, oil and gas industry

## Abstract

This study evaluates the API 650 design procedure for steel storage tanks, incorporating nonlinear dynamic analysis with large deformation effects. Focusing on seismic vulnerability, the case study examines storage tanks proposed for construction in Naples, Italy, assessing their performance under site-specific seismic conditions. A target spectrum and 20 earthquake records were selected to reflect regional seismic characteristics. Initial tank thicknesses were calculated using API 650 guidelines and subsequently analyzed through nonlinear time-history simulations in SAP2000. Results reveal that thicknesses derived from API 650s linear average spectrum equations are insufficient for real seismic demands. Through a trial-and-error methodology, optimal thicknesses were determined to ensure satisfactory performance across all seismic records. Key findings highlight significant variations in mode participation, the frequent occurrence of elephant-foot buckling in tanks with lower H/R ratios, and the limitations of linear spectral analysis for realistic earthquake scenarios. Given the vital role of storage tanks in the oil and gas industry, this study emphasizes the need to integrate nonlinear time history analysis into design processes to enhance seismic resilience, particularly in high-risk regions.

## 1. Introduction

Sloshing is a phenomenon that occurs in fluid-filled containers subjected to external forces such as seismic activity or sudden accelerations [1,2]. In civil engineering, sloshing is particularly relevant in structures such as liquid storage tanks, dams, reservoirs, and even buildings with large water tanks or pools. An earthquake causes the fluid within the structure to slosh back and forth, creating dynamic pressure fluctuations on the walls of the container. These pressure fluctuations can exert significant force on the structure, potentially causing damage or failure if not properly accounted for in the design. The effects of earthquakes on sloshing can be severe, especially in structures with large volumes of fluid [3]. The dynamic forces generated by sloshing can induce additional stresses on the structure, leading to structural instability, cracking, or even collapse. Therefore, it is essential to understand and accurately predict the behavior of sloshing to design structures that can withstand seismic events. Mathematically, the behavior of sloshing can be described by various equations and models, depending on factors such as the shape of the container, the characteristics of the fluid, and the intensity of the external forces. One commonly used approach is to model sloshing as a multiphase fluid-solid interaction problem, where the fluid is treated as a continuous medium subject to the laws of fluid dynamics and the structure is modeled as a solid body subject to structural mechanics principles. The mathematical relationships governing sloshing typically involve equations of motion for both the fluid and the structure, coupled with boundary conditions that account for the interaction between the fluid and the structure. These equations are often nonlinear and may require numerical methods such as finite element analysis or computational fluid dynamics to be solved. Experimental testing and validation are also crucial for understanding and predicting the behavior of sloshing in real-world structures. Researchers use physical models and scale prototypes to simulate sloshing behavior under controlled conditions and validate mathematical models and numerical simulations. In summary, sloshing is a complex phenomenon that can have significant effects on structural integrity, especially during seismic events. Understanding and accurately predicting sloshing behavior requires a combination of mathematical modeling, numerical simulation, and experimental validation [3].

Fluid storage tanks are critical structures in the oil and gas industry, essential for the storage of oil, water, and other substances. Ensuring their safety during and after earthquakes is particularly important in seismically active regions, where the threat of NATECH events (Natural Hazard Triggering Technological Disasters) poses a significant risk of technological catastrophes. Cylindrical liquid storage tanks, commonly used for chemicals, petrochemicals, liquefied natural gas (LNG), and water, are especially vulnerable to seismic forces due to their lower redundancy, ductility, and energy dissipation capacity compared to conventional structures [4]. Failures during earthquakes have been observed in numerous historical events, including the Long Beach earthquake (1933), the 1952 earthquake in California, and the 1960 earthquake in Chile [4]. Other significant events include the Northridge earthquake (1994) and Kobe earthquake (1995), as well as the Silakhor earthquake in 2006 in western Iran [5]. In Italy, the L’Aquila earthquake (2009) and the Emilia earthquake (2012) [6] highlighted vulnerabilities in structural systems. Additionally, the Sedan and Lake Grassmere earthquakes in New Zealand in 2013, with magnitudes of 6.5, and the Kaikōura earthquake in 2016, with a magnitude of 7.8, further demonstrated the devastating impact of seismic events on infrastructure [7]. The Tohoku earthquake in 2011 in Japan caused new damage to tanks on the coast of the Pacific Ocean. Zama et al. [8] conducted a study on the damage of tanks under this earthquake. The Napa earthquake in 2014 also caused damage to tanks in San Francisco Bay [9,10]. Post-earthquake studies in this area showed that the walls of these cylindrical storage tanks faced severe damage and local buckling. In another study in 2011, Korkmaz et al. [11] assessed the risk of Turkish industrial tanks under different earthquakes. To prevent such disasters and mitigate socio-economic and environmental impacts, resilient seismic design is imperative [12,13].

Unlike conventional structures such as buildings and bridges, the dynamic behavior of liquid storage tanks is distinct due to the interaction between the contained liquid and the tank walls. This interaction subjects the tanks to inertial seismic forces and hydrodynamic pressures. Housner’s mechanical model [14] provides a realistic representation of this behavior, dividing the hydrodynamic response into two uncoupled components: the impulsive component, where the lower liquid mass moves with the tank walls, and the convective component, characterized by sloshing in the upper liquid layer. Studies [15,16] have shown that the tank’s overall seismic response is predominantly influenced by the impulsive component. In contrast, the convective component is often negligible due to its longer periods (e.g., over 6 s for the tank studied) compared to the fundamental period of the tank-liquid system (approximately 1.6 s for isolated tanks or less than 0.2 s for fixed-base conditions). Consequently, typical damage modes observed in tanks during past earthquakes, such as “elephant foot” and “diamond shape” buckling, are primarily associated with the impulsive component [17].

In the analysis of fluid-filled tanks, two primary parameters influencing stress are hydrodynamic pressure and sloshing amplitude [18,19,20,21]. The damage and failure of storage tanks during earthquakes have driven researchers to incorporate hydrodynamic pressure into their calculations [22,23]. Housner [14] developed a widely used analytical model to estimate hydrodynamic pressure from horizontal ground motion. Haroun and Tayel [24] extended this approach with a finite element model for dynamic tank response, while Park et al. [25] applied the boundary element method to study rectangular tanks. Chen et al. [26], using a finite difference approach, analyzed fluid motion under harmonic and seismic vibrations. Further studies have addressed various aspects of tank behavior under seismic excitation. Hoskins and Jacobsen [27] provided experimental and analytical relations for rigid rectangular tanks under horizontal loading. Kianoush and Chen [28] utilized the added mass method with iterative solutions to model hydrodynamic pressure in two-dimensional spaces. Veletsos [29] examined seismic responses of fluid tanks on rigid and elastic foundations, while Wu et al. [30] considered viscosity in analytical sloshing studies. More recent efforts have focused on advanced modeling techniques. Estekanchi and Alembagheri [31] used the endurance time method to analyze sloshing phenomena, and Constantine et al. [32] investigated damping due to vertical beaming in single-degree-of-freedom systems. Jamshidi et al. [33] introduced a mathematical model for three-dimensional reservoirs using coupled boundary elements and potential equations. Huang et al. [34] modeled nonlinear sloshing behavior in the time domain, contributing to a deeper understanding of fluid-structure interaction [35,36].

Several methods exist to mitigate damage risks in storage tanks, with two prominent strategies being widely adopted. The first involves increasing the thickness of tank walls and base plates to reduce axial stress and minimize the risk of buckling or base plate rotation. However, this approach can increase seismic energy input and project costs, presenting its own challenges. The second strategy focuses on employing passive control devices, such as dampers and base isolators, which absorb and dissipate seismic energy [37]. These devices effectively reduce the impact of seismic forces, enhancing the structural integrity and resilience of tanks during earthquakes [38].

A comprehensive review of the literature highlights the vulnerability of storage tanks critical for post-seismic operations to damage during earthquakes. Understanding their seismic response is therefore essential. This case study examines the nonlinear behavior of 10 storage tanks under seismic loading, focusing on geometric and material properties. The tanks, with height-to-radius (H/R) ratios ranging from 0.3 to 3, were analyzed using dimensions based on the API 650 [39] standard. The seismic region of Naples, Italy, provided the target spectrum and 20 seismic records for the analysis. Initial tank thicknesses were calculated following Annex E of API 650 and the ASCE 7 [40] target spectrum. These values were then assessed through nonlinear dynamic analysis under various seismic conditions. The results indicate that thicknesses derived from linear equations and averaged spectra may not be universally applicable. An iterative trial-and-error approach was ultimately used to determine the optimal thickness values for enhanced seismic performance.

## 2. Added Mass Method and Verification

For the storage tank with an H/R ratio of 0.3 (the smallest model), three-dimensional fluid modeling posed significant computational challenges due to the complexity of fluid-structure interaction and structural nonlinearity, often leading to analysis divergence. To address this, alternative methods were explored, and the “added mass” method [41] was identified as an effective approach to optimize computational efficiency.

The “added mass” method simplifies fluid-structure interaction by quantifying the additional inertia imparted by fluid motion to the structure during dynamic loading, such as seismic events. This approach considers the structure’s need to accelerate not only its own mass but also a portion of the fluid’s mass, termed “added mass”, which depends on the mode shapes of fluid motion. In cylindrical tanks, this method effectively captures the dominant fluid motion, such as sloshing, providing a computationally efficient means to evaluate seismic behavior:

(1) $$m_{a\;}=\rho V_{added}$$

where ρ is the fluid density and $V_{added}$ (the volume of fluid effectively moving with the containment) for a cylindrical tank, can be approximated using empirical or analytical methods. In the simplest form, for the first mode of sloshing, the added mass can be estimated as:

(2) $$m_{a}\;=C\rho \pi R^{2}h$$

where *C* is a dimensionless coefficient that depends on the aspect ratio of the tank (height to radius ratio) and the mode of sloshing.

The liquid is modeled using an added mass approach, in which the mass is obtained from a pressure distribution for the impulsive mode of a tank-liquid system originally developed by Veletsos and Shivakumar [41]. This pressure distribution is due to the rigid body horizontal motion of a rigid tank-liquid system and is described as:

(3) $$P_{i}(\eta ,\theta ,t)=c_{i}(\eta )\rho R{\ddot{x}}_{g}(t)\cos\theta$$

where *P_i_* is the impulsive pressure; η is a non-dimensional vertical coordinate and is equal to *z/H_L_*; *z* is the vertical coordinate measured from the tank bottom; *R* is the tank radius; ${\ddot{x}}_{g}(t)$ is the ground acceleration; and *t* is the time. The function *c_i_* (η) defines the impulsive pressure distribution along the cylinder height and is computed as:

(4) $$c_{i}(\eta )=1-\sum_{n=1}^{\infty }c_{cn}(\eta )$$

where $c_{cn}(\eta )$ is a function that defines the convective pressure distribution along the cylinder height and takes the form:

(5) $$c_{cn}(\eta )=\frac{2}{\lambda_{n}^{2}-1}\frac{\cosh(\lambda_{n}(H/R)\eta )}{\cosh(\lambda_{n}(H/R))}$$

The parameter $\lambda_{n}$ is the *n*^st^ root of the first derivative of the Bessel function of the first kind and first order. The first three roots are $\lambda_{1}$ = 1.841, $\lambda_{2}$ = 5.311, and $\lambda_{3}$ = 8.536. The function *c_i_* (η) converges rapidly with the number of terms in the summation in Equation (4), and thus it is sufficient to include three coefficients $c_{cn}$.

The mass distribution in the height of the structure is obtained from Equation (6), where η indicates the coordinate along the axis of the cylinder, ρ is the water density, *A_element_* is area of element, and the function $c_{i}(\eta )$ describes the pressure distribution along the height of the storage tank and can be determined after Veletsos and Shivakumar [41]:

(6) $$m_{i}=c_{i}\times \rho \times R\times A_{element}$$

**Figure 1:**
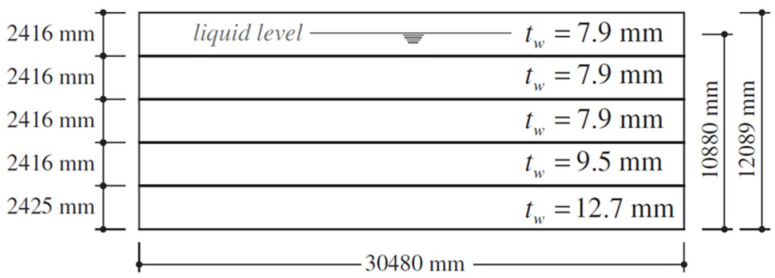
Geometric characteristics of the tank considered as a case studied.

**Figure 2:**
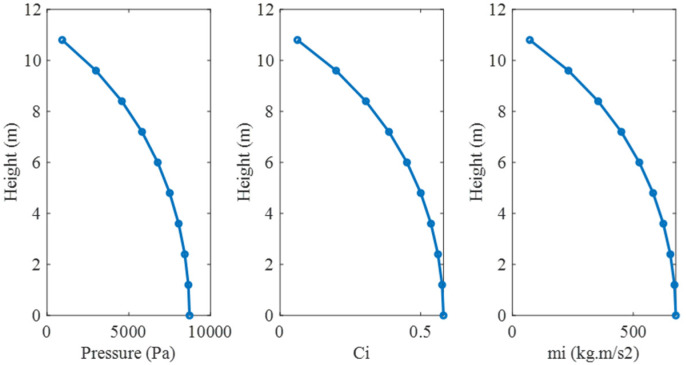
Quantitative distribution of pressure, parameter $C_{i}$, and mass.

In this method, to consider the hydrodynamic effects of the fluid, the mass of the fluid is concentrated on the nodes. In this regard, first for the accuracy of the modeling, a storage tank from [42] was selected, and the frequency of the first mode and the shape of the first mode were examined. Figure 1 shows the geometric characteristics of the investigated storage tank, and Figure 2 shows the distribution of pressure, mass, and parameter $C_{i}$ at the height of the investigated tank based on the calculations.

**Figure 3:**
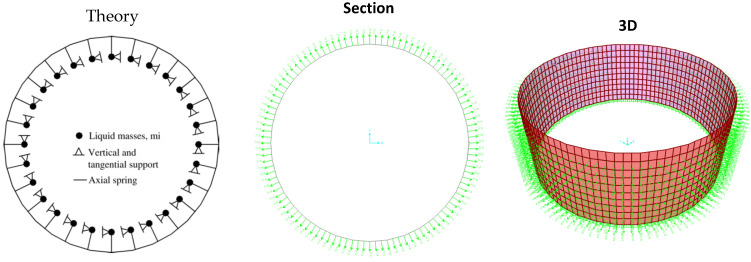
Model with normal mass around the circumference using SAP2000 v10 software.

The added liquid mass in lumped form is attached to the shell nodes by means of massless spring elements considered as rigid links, as shown in Figure 3. The one-direction springs have supports oriented in their local axes that constrained the motion of the nodal masses to the normal direction of the shell. The motions of each support are restricted in the global tangential direction (i.e., perpendicular to the element axis) and in the vertical direction, whereas it is free to move in the radial direction (i.e., local axial direction of the spring). Liquid masses can only move in the radial direction.

In Figure 3, the model built using the added mass method using SAP2000 is illustrated, and Table 1 presents the calculated natural periods. Due to the symmetry of the steel storage tank, which results in duplicate natural modes, only the odd natural periods are listed. In Table 1, *n* denotes the number of circumferential waves. Based on the analysis conducted in SAP2000, the natural period of the structure’s first mode is approximately 2.73 s, differing by less than 0.5% from the value reported in reference [42]. Additionally, Table 1 indicates that the parameter *n* in SAP2000 is also 14. Therefore, these results confirm the reliability of the modeling process.

## 3. Hoop and Longitudinal Stresses in Cylindrical Storage Tanks

Cylindrical storage tanks, such as those used for storing fluids under pressure, experience various stresses due to the internal pressure exerted by the fluid, inducing various types of damages [2,6,43]. The primary stresses in a cylindrical storage tank are hoop (circumferential) stress and longitudinal (axial) stress.

### 3.1. Hoop (Circumferential) Stress

Hoop or circumferential stress is the stress exerted in the circumferential direction of the cylindrical wall. This stress acts perpendicular to the axis of the cylinder and is often the most significant stress in a cylindrical storage tank. For a thin-walled cylindrical storage tank with internal pressure *P*, internal radius *r*, and wall thickness *t* (where *t* << *r*), the hoop stress $\sigma_{h}$ is given by:

(7) $$\sigma_{h}=\frac{P\times r}{t}$$

### 3.2. Longitudinal (Axial) Stress

Longitudinal stress is the stress exerted along the axis of the cylindrical storage tank. This stress is parallel to the axis of the cylinder and is typically lower than the hoop stress. For a thin-walled cylindrical tank, the longitudinal stress $\sigma_{l}$ is given by:

(8) $$\sigma_{l}=\frac{P\times r}{2t}$$

**Figure 4:**
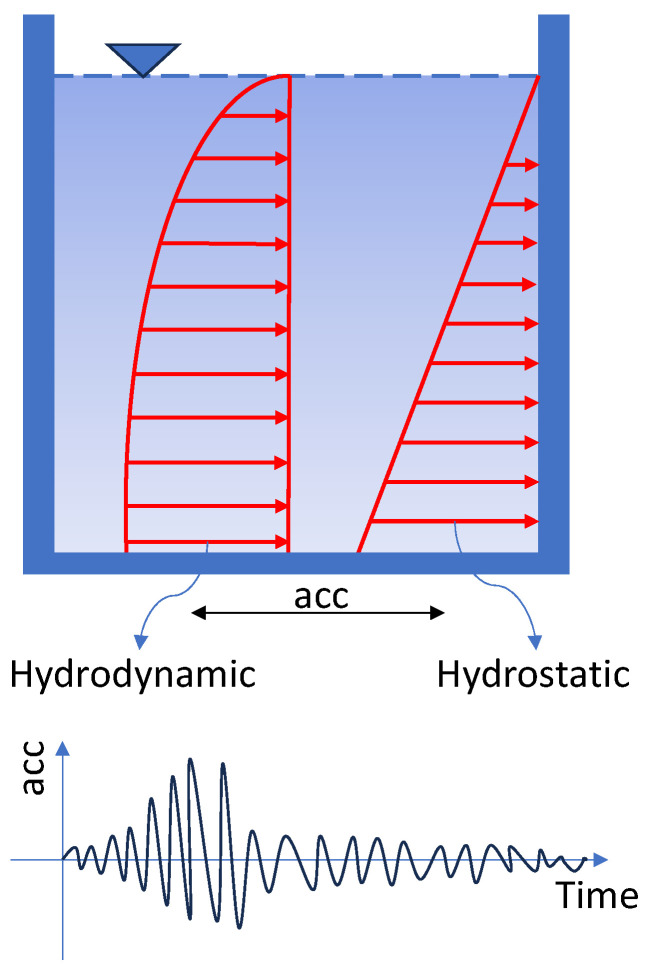
Hydrostatic and hydrodynamic distribution of fluid in tanks.

Equations (7) and (8) are related to static analysis; when a tank containing fluid is subjected to a seismic load, the pressure distribution is applied hydrodynamically. Figure 4 shows the hydrostatic and hydrodynamic distribution of fluid in storage tanks.

API 650 provides criteria for determining stresses resulting from hydrodynamic pressure. According to these criteria, hoop and longitudinal stresses are calculated based on the design spectrum of ASCE 7.

(9) $$N_{i}=\left\{\begin{array}{cc} 8.48A_{i}GDH[\frac{Y}{H}-0.5{\left(\frac{Y}{H}\right)}^{2}]\tanh(0.866\frac{D}{H}) & D/H\geq 1.33\\ 5.22A_{i}GD^{2}[\frac{Y}{0.75D}-0.5{\left(\frac{Y}{0.75D}\right)}^{2}] & D/H<1.33\&Y/D<0.75\\ 2.6A_{i}GD^{2} & D/H<1.33\&Y/D\geq 0.75 \end{array}\right.$$

(10) $$N_{c}=\frac{1.85A_{c}GD^{2}\cosh[\frac{3.68(H-Y)}{D}]}{\cosh[\frac{3.68H}{D}]}\;\;\;\;\;\;\;\;\;\;\;\;\;\;\;\;\;\;\;\;\;\;\;\;\;For\;all\;D/H$$

where $A_{i}\;$ and $A_{c}\;$ are the impulsive and convective spectral accelerations, respectively; $N_{i}$ is the impulsive hoop membrane force in the storage tank shell, $N_{c}\;$ is the convective hoop membrane force in the tank shell, G  is the specific gravity, and Y  is the distance from the liquid surface to the analysis point. The dynamic hoop tensile stress should be directly combined with the product hydrostatic design stress in determining the total stress:

(11) $$\sigma_{T}=\sigma_{h}\pm \sigma_{s}=\frac{N_{h}\pm \sqrt{N_{i}^{2}+N_{c}^{2}+{\left(A_{v}N_{h}\right)}^{2}}}{t}$$

where $\sigma_{T}$ is the total stress, while $\sigma_{h}$ and $\sigma_{s}$ are the product hydrostatic the hoop stress in the tank and hoop stress in the shell tank due to impulsive and convective forces of the stored liquid, respectively. $N_{h}$ is the product hydrostatic membrane; $A_{v}$ is the vertical spectral acceleration; and *t* is the thickness of the shell ring under consideration.

The maximum longitudinal shell compression stress at the bottom of the shell when there is no calculated uplift, *J* ≤ 0.785, shall be determined by the formula:

(12) $$\sigma_{c}=(w_{t}(1+0.4A_{v})+\frac{1.273M_{rw}}{D^{2}})\frac{1}{1000t}$$

The maximum longitudinal shell compression stress at the bottom of the shell when there is calculated uplift, *J* > 0.785, shall be determined by the formula:

(13) $$\sigma_{c}=(\frac{w_{t}(1+0.4A_{v})-w_{a}}{0.607-0.18667{\left(J\right)}^{2.3}}-w_{a})\frac{1}{1000t}$$

where $w_{t}$ is the weight of the roof and wall of the storage tank acting at the base of the shell; $w_{a}$ is force resisting uplift in the annular region; and *J* is the anchorage ratio.

In this research, the hoop and longitudinal stress results obtained from API 650 will be compared with the results obtained from non-linear models (materials and geometry). Furthermore, the stresses obtained from the non-linear dynamic analysis are compared with the allowable stress value suggested by API 650. Allowable stress for longitudinal and hoop stress is obtained from Equations (14) and (15), respectively:

(14) $$F_{allow}^{Long}=\left\{\begin{array}{cc} \frac{83t}{D} & \frac{GHD^{2}}{t^{2}}\geq 44\\ \frac{83t}{2D}+7.5\sqrt{GH}<0.5Fy & \frac{GHD^{2}}{t^{2}}<44 \end{array}\right.$$

(15) $$F_{allow}^{Hoop}=\min(1.33S_{d},0.9Fy)$$

where *t* is tank thickness; *D* is tank diameter; *H* is fluid height; *G* is design specific gravity; *S_d_* is basic allowable membrane; and *Fy* is yield strength.

## 4. Selected Seismic Records

**Figure 5:**
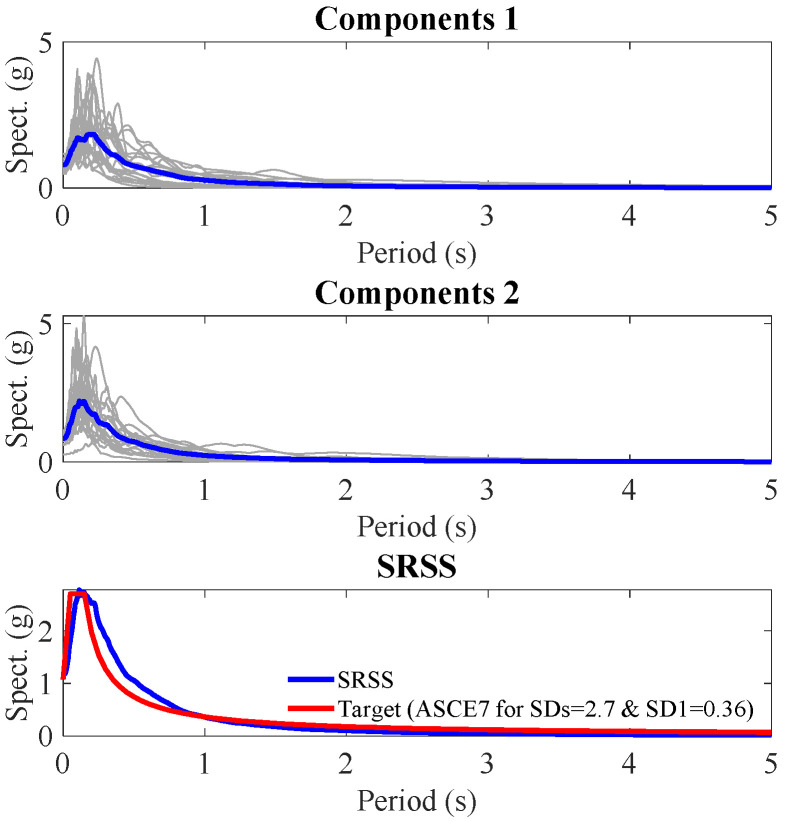
Mean spectrum of each component of the ground motions.

In this case study, 20 ground motion records were selected based on site-specific characteristics. To develop the design spectrum, the response spectrum for each horizontal component (two orthogonal directions) of each ground motion record was first obtained in accordance with the method outlined in ASCE 7. The SRSS (Square Root of the Sum of the Squares) spectrum for each record was then computed by combining the two perpendicular horizontal components. Subsequently, the average SRSS spectrum for the 20 seismic records was used to derive a design spectrum using the ASCE 7 provisions. The design spectrum is influenced by two key parameters, SDs (spectral response acceleration at short periods) and SD1 (spectral response acceleration at a 1-s period). The values of these parameters were iteratively determined as 2.7 and 0.36, respectively, to ensure that the derived design spectrum closely matched the average SRSS spectrum. The mean spectrum of each component and the mean spectrum of SRSS are shown in Figure 5.

## 5. Investigation of the Storage Tanks Designed According to Annex E of API 650

**Figure 6:**
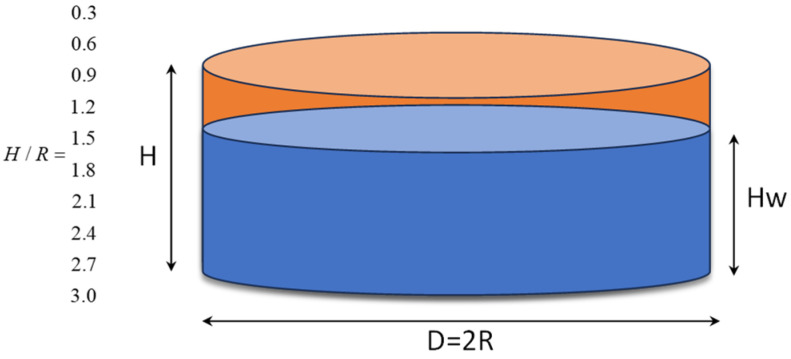
Schematic 3D representation of the tank analyzed in various configurations.

In this research, 10 cylindrical storage tanks were investigated. It is assumed that 80% of the volume of tanks is filled with fluid. The investigated tanks have H/R ratio equal to 0.3, 0.6, 0.9, 1.2, 1.5, 1.8, 2.1, 2.4, 2.7, and 3. The material used in the storage tanks is considered A283c grade c steel. In Figure 6, the schematic shape of the storage tanks is illustrated. According to Section 5.6.1.1 of API 650-2021 [39] and based on the diameter of the tanks used in this research, the recommended minimum thickness is equal to 6 mm. On the other hand, the minimum thickness for the storage tanks has been chosen in such a way that the ratio of the stress demand in the structure (Equations (11)–(13)) to the capacity stress (Equations (14) and (15)) is around 1.

The demand-to-capacity ratio (DCR), calculated using the equations outlined in Section 3 and the target spectrum depicted in Figure 5, is summarized in Table 2. This Table 2 presents a comprehensive overview of each storage tank’s geometry, the corresponding allowable stresses, and the DCR values for both hoop and longitudinal stresses, as determined by the API 650 design methodology.

### 5.1. Spectral Analysis of Storage Tanks Designed According to API 650

Prior to performing the nonlinear time history analysis (NTHA) on the storage tanks, a comparison was made between the results obtained from API 650 and those derived from the spectral analysis, in accordance with the ASCE 7 target spectrum. For this comparison, all storage tanks were modeled according to the geometry presented in Table 2 and analyzed using the target spectrum shown in Figure 5. The added mass method was employed to account for the effects of fluid-structure interaction. Since spectral analysis is a type of dynamic analysis with a linear nature, a behavior coefficient was applied to account for nonlinearity in the analysis. API 650 recommends two coefficients for these storage tanks: $R_{wc}$ = 2 and $R_{wi}$ = 3.5. In this research, the investigated tanks have been analyzed once for $R_{wc}$ = 2 and once for $R_{wi}$ = 3.5. Table 3 shows the results for $R_{wc}$ = 2 while Table 4 shows the results for $R_{wi}$ = 3.5. As seen in Table 3, Table 4 and Table 5, when the behavior coefficient is set to 2, the demand-to-capacity ratio (DCR) for longitudinal stress exceeds 1 in 70% of the tanks.

**Figure 7:**
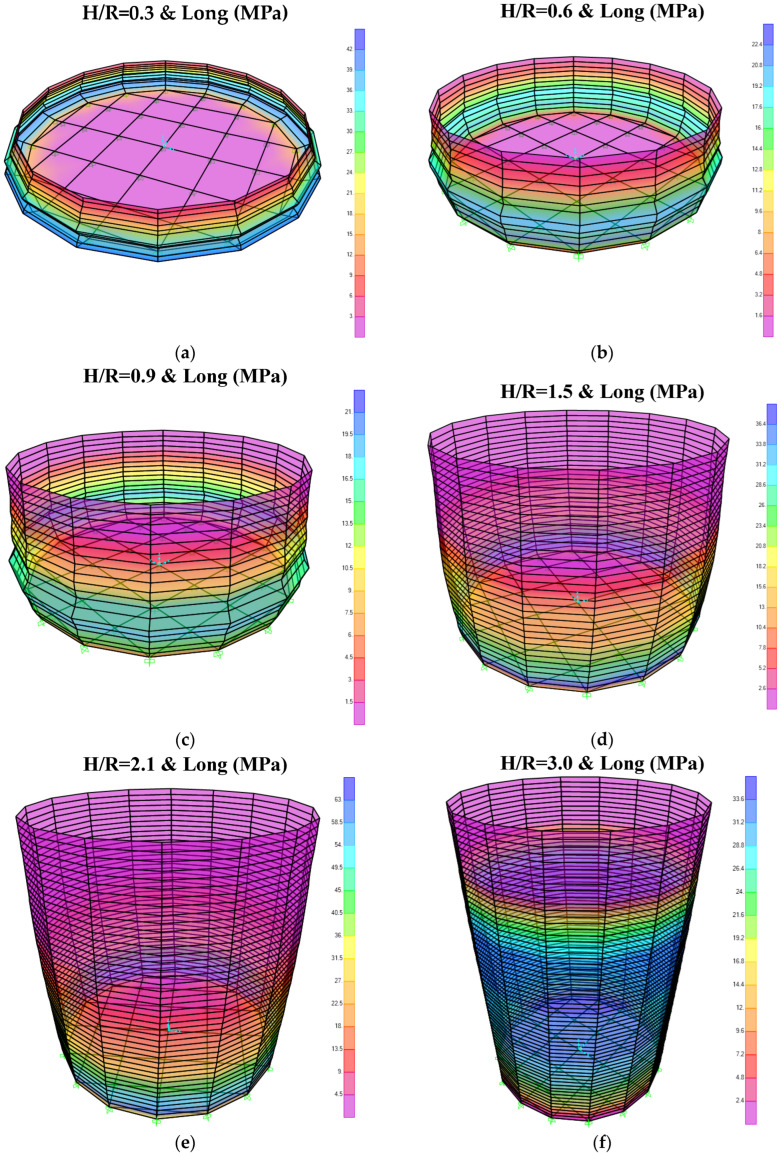
Longitudinal stress contour obtained from spectral analysis for behavior coefficient equal to 2: (**a**) *H/R* = 0.3; (**b**) *H/R* = 0.6; (**c**) *H/R* = 0.9; (**d**) *H/R* = 1.5; (**e**) *H/R* = 2.1; (**f**) *H/R* = 3.0.

As an example, Figure 7 illustrates the longitudinal stress contours for various tanks with a behavior coefficient of 2. Notably, elephant-foot buckling is distinctly evident in the tanks shown in Figure 7a–c.

### 5.2. Time History Analysis of the Storage Tanks Designed Based on API 650

**Figure 8:**
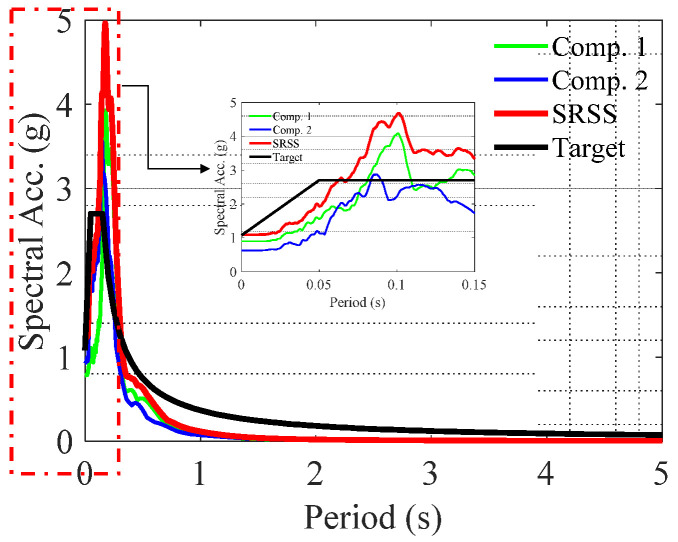
Earthquake spectrum for seismic record No. 18.

In the previous section, it was shown that the storage tanks designed in accordance with the API 650 and target spectrum of ASCE 7 were consistent with the outcomes derived from the spectral analysis of the structure. Nonetheless, it is crucial to consider that spectral analysis relies on the average spectrum of earthquake records and is inherently a linear analysis. On the other hand, the fundamental period of the evaluated tanks, whose geometry is derived from Annex A of API 650, is less than 0.15 s. Regarding the spectra in Figure 8, it is evident that for certain earthquake records, such as record E18, the SRSS (Square Root of the Sum of the Squares) spectrum exceeds the target spectrum by up to three times for periods below 0.15 s. Therefore, it can be predicted that structures designed based solely on the target spectrum may not exhibit adequate performance when subjected to seismic records during the time history analysis.

In this section, storage tanks designed according to Annex E of API 650 were subjected to nonlinear time history analysis, accounting for both material and geometric nonlinearity, under various site-specific earthquake records.

In Table 6, the maximum displacement for each seismic record and each storage tank is presented individually. When maximum displacement is considered as the controlling parameter, it is observed that the critical seismic records—those leading to the maximum displacement in a tank—include records E8, E12, E14, E18, and E20. As specified by ASCE 7, the behavior factor (*R*) for storage tanks is 3. By comparing the results of nonlinear time history analysis with those obtained from spectral analysis using *R* = 3, the ratios presented in the last row of Table 5 are achieved. Additionally, the final two rows of Table 6 provide the average displacement across various records and the ratio of this average displacement to the displacement derived from spectral analysis. These results indicate that the ratio of average displacement to spectral displacement ranges from 2.61 to 4.64. Consequently, it can be inferred that the target spectrum, as applied to the analyzed tanks, does not serve as a reliable criterion for design. Given the critical role of storage tanks, it is recommended that the design process for this category of structures be conducted using time history analysis to ensure accuracy and safety.

**Figure 9:**
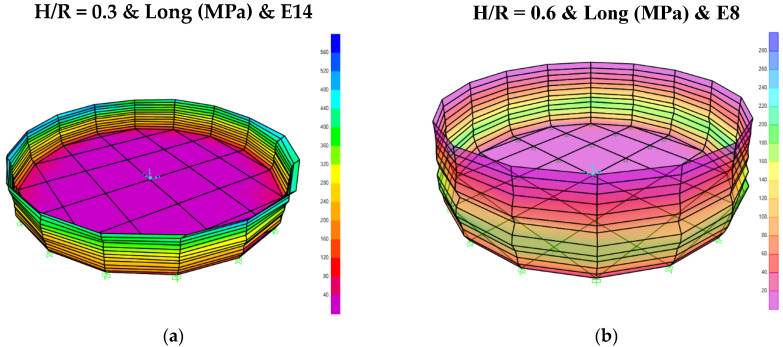

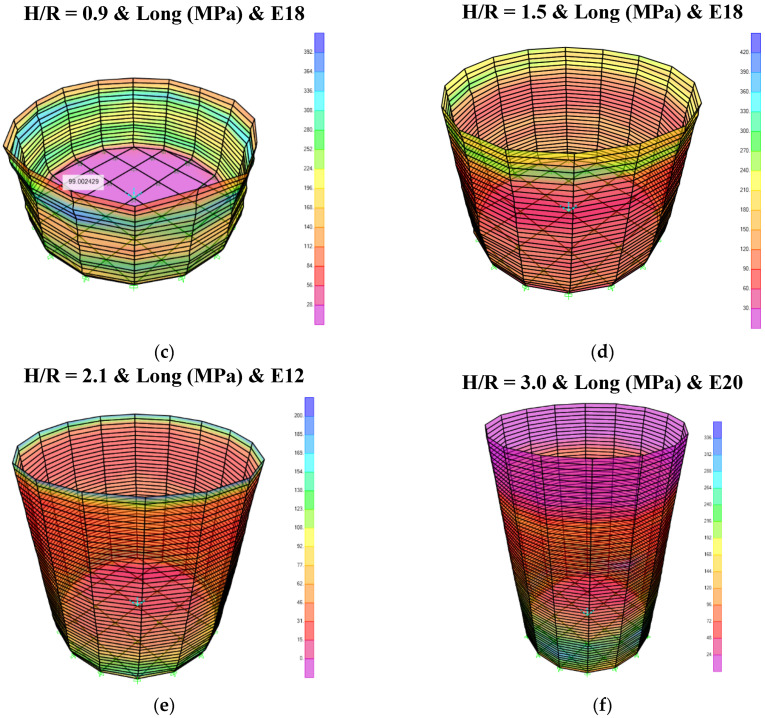
Longitudinal stress contour for tanks designed according to API 650 for critical earthquake: (**a**) *H/R* = 0.3; (**b**) *H/R* = 0.6; (**c**) *H/R* = 0.9; (**d**) *H/R* = 1.5; (**e**) *H/R* = 2.1; (**f**) *H/R* = 3.0.

Figure 9 illustrates the longitudinal stresses corresponding to the critical earthquake for each storage tank, as identified in Table 6. As observed, the intensity of the earthquakes significantly exceeded the structural capacity of the tanks, resulting in frequent plastic deformations at their edges.

Table 7 presents the DCR for longitudinal and hoop stresses under critical earthquake conditions for various storage tanks. It is evident that longitudinal stress is the more critical parameter across all tanks. Specifically, the DCR for hoop stress consistently remains below 1.5, whereas the DCR for longitudinal stress ranges from 3.5 to 27. These findings indicate that the demand for longitudinal stress, as calculated using the target spectrum specified by ASCE 7 and API 650, significantly underestimates the results obtained from critical earthquake scenarios.

**Figure 10:**
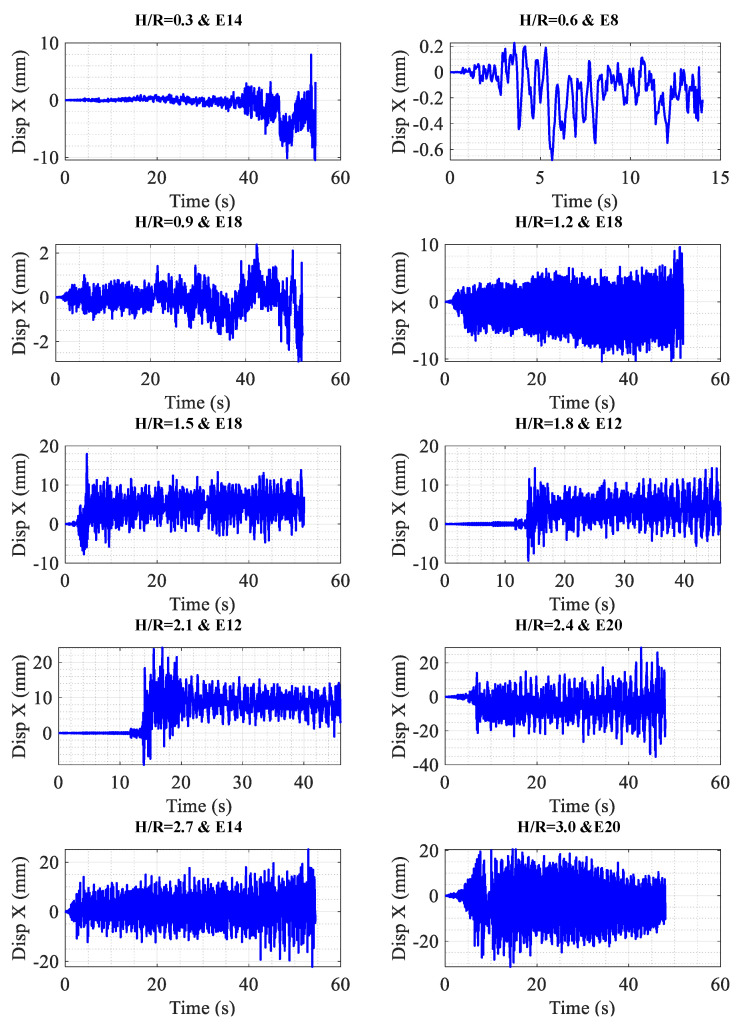
Upper edge displacement for critical earthquakes in different tanks.

To demonstrate the nonlinear behavior of the storage tanks, the displacement of a node located at the upper edge is depicted in Figure 10. As shown in Figure 10, permanent displacements have developed in all tanks, indicating that they have entered the plastic deformation range under the critical seismic records. Very recent research on the application of advanced numerical models using NLTH analysis can be found here [44,45,46].

## 6. Optimum Design of Storage Tanks Through Nonlinear Time History Analysis

In the preceding section of this study, the appropriate thickness for the storage tanks was determined using the methodology outlined in Annex E of API 650. However, as the procedure in the aforementioned annex is based on the average spectrum and linear relationships, it does not yield a thickness capable of withstanding all earthquake records. Consequently, in the previous analysis, the storage tank was first evaluated spectrally using the target spectrum specified in ASCE 7. The results of the spectral analysis indicated that the structure possessed adequate strength. Nonetheless, when nonlinear time history analysis was conducted for the 20 site-specific earthquake records under investigation, it was observed that the DCR exceeded 20 for some records. This discrepancy can be attributed to two primary factors: (1) for periods shorter than 0.15 s, the spectral content of certain earthquake records exceeded the ASCE 7 target spectrum by up to three times, and (2) the time history analysis accounted for both geometric and material nonlinearities, which revealed additional demands on the structure not captured by linear analysis methods.

**Figure 11:**
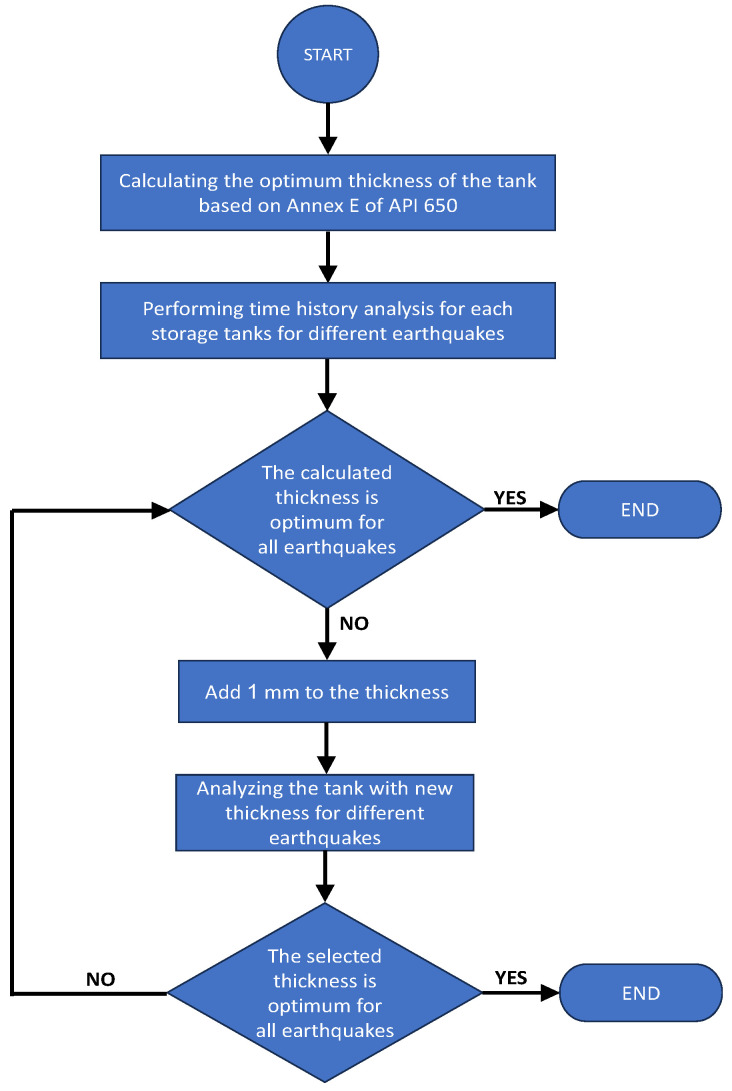
The design flowchart algorithm employed to achieve the optimum thickness.

In this section, the optimal thickness for the storage tanks is determined through a trial-and-error approach involving the evaluation of various thicknesses to ensure that the DCR for the critical earthquake record (the earthquake inducing the highest stress in the structure) is approximately 1. The design algorithm employed to achieve the optimal thickness is illustrated in Figure 11. To accomplish this, over 1000 h of computational analysis were conducted using a system equipped with a 12th-generation Intel i7 processor, leveraging the trial-and-error methodology to achieve the optimum results.

Table 8 presents the optimum thickness values determined by the algorithm shown in Figure 11 for various storage tanks. In all cases, the DCR for longitudinal stress served as the governing factor. The thicknesses were optimized to ensure that the maximum DCR falls within the range of 0.74 to 1. To maintain practicality in engineering and construction scope of work, the thickness values were rounded to the nearest whole millimeter. As shown in Table 8, the tank with a H/R of 2.4 exhibits the lowest DCR compared to the other tanks. It is important to highlight that if the thickness for this storage tank is set to 47 mm, the DCR would increase to approximately 1.3.

**Figure 12:**
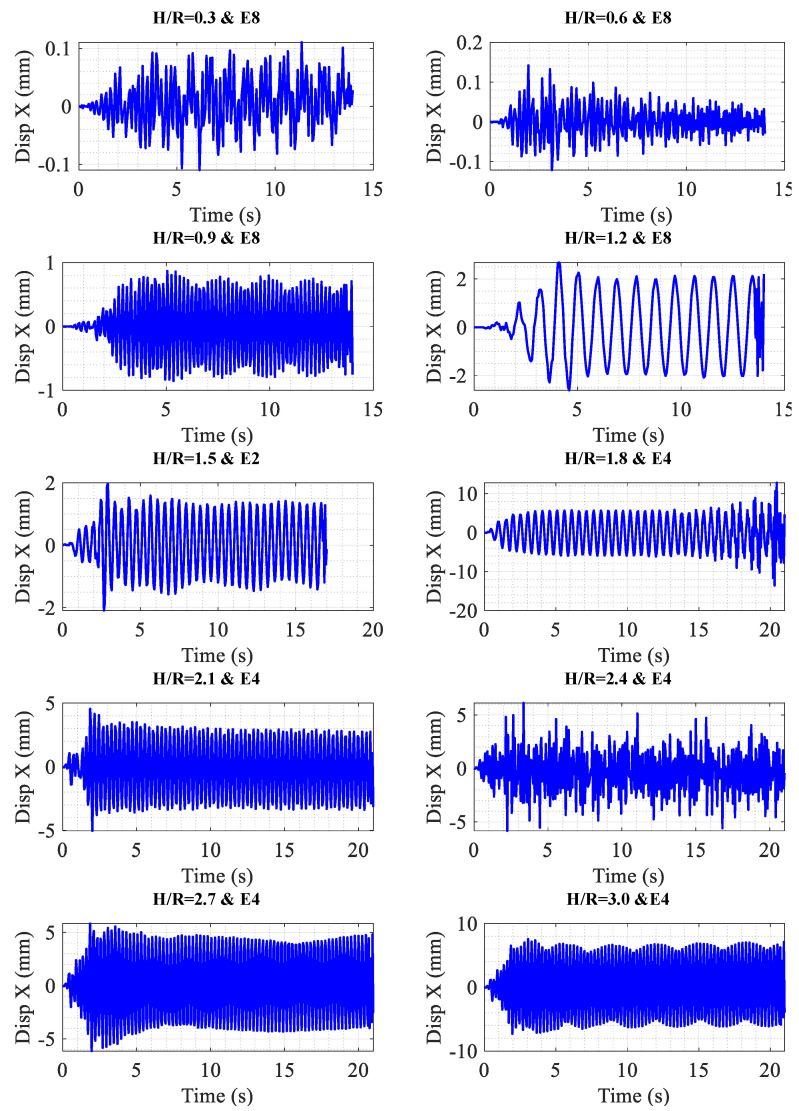
Upper edge displacement for critical earthquakes in different tanks (tank with optimum thickness).

Table 9 presents the maximum displacement for each storage tank under various earthquake scenarios once the nonlinear time history analysis has been applied. From the data in Table 9, it can be figured out that the critical earthquakes, in the state where the storage tanks have not yet entered the nonlinear range (as also depicted in Figure 12), are among earthquakes E2, E4, and E8. This can be anticipated by analyzing the spectra of these earthquakes. Regarding Table 6, which displays the maximum displacement of each storage tank for the target spectrum of ASCE 7 with the behavior coefficient of *R* = 3, if the maximum displacement is taken as the controlling parameter for the storage tanks and the values presented in this table are considered the maximum allowable displacements, the ratio of the average displacement obtained from the nonlinear time history analysis (NLTHA) to the allowable displacement is provided in the last row of Table 9. As shown, this ratio consistently remains less than or equal to 1.

Furthermore, Figure 12 presents the displacement response of the upper edge for the critical earthquake in various storage tanks with optimum thicknesses. As illustrated in Figure 12, the upper edge displacement in all optimized storage tanks oscillates uniformly around zero, indicating the absence of permanent displacement. Therefore, it can be concluded that the behavior of these tanks remains linear during seismic vibrations.

## 7. Conclusions

In this case study, for the first time, a comprehensive review of the design procedure outlined in the API 650 guideline was conducted. To perform that, first, a target spectrum of ASCE 7 and 20 earthquake records was selected according to the site under investigation, Naples, Italy. Based on the selected ASCE 7 target spectrum and the geometry of the storage tanks, the initial thicknesses for each tank were determined using manual calculations from Annex E of API 650. Subsequently, all storage tanks were modeled in SAP2000. Prior to performing the nonlinear time history analysis, a linear spectral analysis was carried out in accordance with ASCE 7. Following this, the storage tanks designed based on Annex E of API 650 were subjected to nonlinear time history analysis.

When it was determined that the thicknesses obtained from Annex E of API 650 were not appropriate for the storage tanks intended for the upcoming project, which is a tank farm in Naples, Italy, an optimum thickness for each storage tank was determined using a trial-and-error process, considering all site-specific earthquakes. The results of this analysis are presented as follows:

- The storage tanks with smaller H/R ratios, designed in accordance with Annex E of API 650, exhibit the “elephant-foot” buckling phenomenon in their fundamental mode shape. This behavior is particularly noticeable in storage tanks with H/R ratios of 0.3, 0.6, and 0.9. As the H/R ratio increases, the fundamental mode shape transitions, increasingly resembling the behavior of a cantilever column.
- The analysis of tanks designed in accordance with Annex E of API 650 reveals that longitudinal stress is the governing factor in all cases. The study indicates that the DCR for hoop stress consistently remains below 1.5, whereas the DCR for longitudinal stress ranges from 3.5 to 27. These findings suggest that the longitudinal stress demand derived from the target spectrum and API 650 design procedure significantly underestimates the critical earthquake demands resulted from the nonlinear time history analysis.
- For storage tanks with optimum thickness, DCR for longitudinal stress has been the controlling factor in all cases. The optimum thickness for each tank was selected such that the maximum DCR ranged between 0.74 and 1. Given that the thickness was selected to ensure practicality in industrial applications, the final thickness was reported as an integer in millimeters.
- For storage tanks with optimum thickness, it can be observed that the critical earthquake, under conditions where the structure remains within the linear range, corresponds to records E2, E4, and E8. This outcome could have been anticipated by analyzing the spectra of these seismic records. When the maximum displacement is considered as the controlling parameter, and the displacement values presented in Table 5.5 are regarded as the maximum allowable displacements, the ratio of the average displacement to the allowable displacement demonstrates that this ratio consistently remains less than or equal to 1.
- The analysis of storage tanks designed based on Annex E of API 650 shows that while the design aligns properly with the target spectrum of ASCE 7 resulting from spectral analysis, there are notable limitations. Spectral analysis relies on the average earthquake spectrum and is inherently linear, whereas real earthquake records exhibit significant deviations. Specifically, for fundamental periods less than 0.15 s, certain earthquake records, like record number 18, demonstrate that the SRSS spectrum can exceed the target spectrum of ASCE 7 by up to three times. This discrepancy indicates that structures designed solely on the target spectrum of ASCE 7 may not perform adequately under actual seismic events. Nonlinear analysis of materials and geometry for various seismic records highlights that the maximum displacements for different tanks vary significantly depending on the earthquake record. The critical earthquakes leading to maximum displacements include records 8, 12, 14, 18, and 20. When comparing the nonlinear time history analysis results with spectral analysis for *R* = 3, the displacement ratios reveal that the target spectrum of ASCE 7 does not provide a suitable design criterion for the investigated tanks. Therefore, it is recommended that, given the critical nature of storage tank structures, especially in the oil and gas industry, the design process should incorporate nonlinear time history analysis to ensure structural integrity and performance during actual seismic events.

## Figures and Tables

**Table 1:** Periods, *T*, of the first 30 natural modes; *m* and *n* indicate the number of axial and circumferential waves.

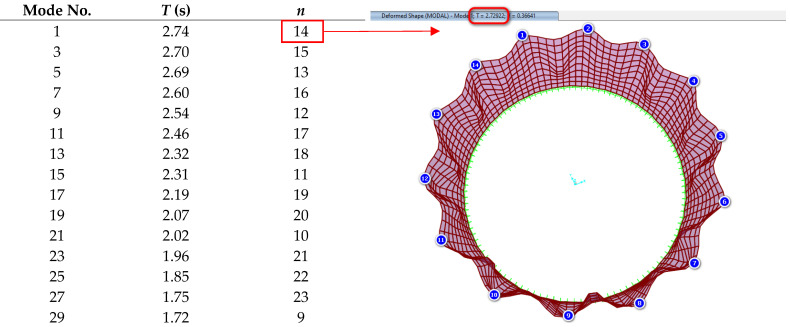

**Table 2:** A summary of specifications of the investigated tanks.

No.	H/R	R (m)	$H_{w}$ (m)	H (m)	Thick_min_ (mm)	$F_{allow}^{Long}$ (MPa)	$F_{allow}^{Hoop}$ (MPa)	DCR_Long_	DCR_Hoop_
1	0.3	12	3.0	3.6	6	20.68	183.81	0.045	0.503
2	0.6	6	3.0	3.6	6	29.52	183.81	0.055	0.251
3	0.9	6	4.5	5.4	6	32.44	183.81	0.146	0.381
4	1.2	6	6.0	7.2	6	34.90	183.81	0.295	0.504
5	1.5	6	7.5	9	6	37.06	183.81	0.497	0.619
6	1.8	6	9.0	10.8	6	39.02	183.81	0.748	0.726
7	2.1	6	10.5	12.6	8	46.34	183.81	1.092	0.583
8	2.4	6	12.0	14.4	10	53.53	183.81	1.017	0.514
9	2.7	6	13.5	16.2	12	60.62	183.81	0.969	0.469
10	3.0	6	15.0	18	14	67.63	183.81	0.937	0.436

**Table 3:** Spectral analysis results for $R_{wc}$ = 2.

No.	H/R	Thick_min_ (mm)	$F_{allow}^{Long}$ (MPa)	$F_{allow}^{Hoop}$ (MPa)	F_Long_ (MPa)	F_Hoop_ (MPa)	Max. Disp (mm)	DCR (Long)	DCR (Hoop)
1	0.3	6	20.68	183.81	44.843	76.894	6.432	**2.17**	0.42
2	0.6	6	29.52	183.81	23.399	43.326	1.881	0.79	0.24
3	0.9	6	32.44	183.81	21.124	82.313	3.794	0.65	0.45
4	1.2	6	34.90	183.81	22.537	66.307	4.102	0.65	0.36
5	1.5	6	37.06	183.81	38.725	73.231	6.121	**1.04**	0.40
6	1.8	6	39.02	183.81	58.894	76.279	9.186	**1.51**	0.41
7	2.1	8	46.34	183.81	64.999	54.507	10.950	**1.40**	0.30
8	2.4	10	53.53	183.81	71.674	44.568	13.455	**1.34**	0.24
9	2.7	12	60.62	183.81	78.865	38.108	16.505	**1.30**	0.21
10	3.0	14	67.63	183.81	86.304	33.437	20.153	**1.28**	0.18

**Table 4:** Spectral analysis results for $R_{wc}$ = 3.5.

No.	H/R	Thick_min_ (mm)	$F_{allow}^{Long}$ (MPa)	$F_{allow}^{Hoop}$ (MPa)	F_Long_ (MPa)	F_Hoop_ (MPa)	Max. Resultant Disp (mm)	DCR (Long)	DCR (Hoop)
1	0.3	6	20.68	183.81	25.624	43.94	3.676	**1.24**	0.24
2	0.6	6	29.52	183.81	13.371	24.758	1.075	0.45	0.13
3	0.9	6	32.44	183.81	12.071	47.036	2.168	0.37	0.26
4	1.2	6	34.90	183.81	12.878	37.890	2.344	0.37	0.21
5	1.5	6	37.06	183.81	22.129	41.846	3.498	0.60	0.23
6	1.8	6	39.02	183.81	33.653	43.588	5.249	0.86	0.24
7	2.1	8	46.34	183.81	37.142	31.147	6.259	0.80	0.17
8	2.4	10	53.53	183.81	40.957	25.467	7.689	0.77	0.14
9	2.7	12	60.62	183.81	45.066	21.776	9.431	0.74	0.12
10	3.0	14	67.63	183.81	49.316	19.107	11.516	0.73	0.10

**Table 5:** Spectral analysis results for R = 3 (ASCE 7).

No.	H/R	Thick_min_ (mm)	$F_{allow}^{Long}$ (MPa)	$F_{allow}^{Hoop}$ (MPa)	F_Long_ (MPa)	F_Hoop_ (MPa)	Max. Resultant Disp (mm)	DCR (Long)	DCR (Hoop)
1	0.3	6	20.68	183.81	29.89	51.26	4.29	**1.45**	0.28
2	0.6	6	29.52	183.81	15.60	28.88	1.25	0.53	0.16
3	0.9	6	32.44	183.81	14.08	54.88	2.53	0.43	0.30
4	1.2	6	34.9	183.81	15.02	44.21	2.73	0.43	0.24
5	1.5	6	37.06	183.81	25.82	48.82	4.08	0.70	0.27
6	1.8	6	39.02	183.81	39.26	50.85	6.12	1.01	0.28
7	2.1	8	46.34	183.81	43.33	36.34	7.30	0.94	0.20
8	2.4	10	53.53	183.81	47.78	29.71	8.97	0.89	0.16
9	2.7	12	60.62	183.81	52.58	25.41	11.00	0.87	0.14
10	3.0	14	67.63	183.81	57.54	22.29	13.44	0.85	0.12

**Table 6:** Maximum displacement obtained from time history analysis for tanks designed according to API 650.

E	Disp. for H/R (mm)									
	0.3	0.6	0.9	1.2	1.5	1.8	2.1	2.4	2.7	3.0
1	9.35	1.26	3.08	4.59	8.64	14.84	12.91	13.23	16.98	14.83
2	14.86	4.51	8.40	12.91	13.29	23.50	29.49	35.50	39.63	32.73
3	12.32	4.01	9.80	12.09	14.19	12.45	13.41	19.13	21.38	52.40
4	34.75	7.07	11.20	14.90	21.58	32.96	37.69	40.61	41.38	55.19
5	10.84	3.77	7.70	6.53	10.75	17.62	19.98	34.02	41.69	57.59
6	19.56	4.25	5.60	6.71	22.67	27.59	36.13	35.01	40.76	57.76
7	22.27	4.61	7.70	15.16	18.04	28.79	26.60	34.82	37.77	44.95
8	13.38	8.17	11.90	15.47	17.94	36.18	38.27	37.94	50.30	69.71
9	10.20	4.19	4.90	7.03	11.79	18.89	19.93	23.85	20.56	27.92
10	17.40	1.70	4.20	12.15	20.11	31.33	35.50	42.36	42.94	45.19
11	16.79	4.28	9.80	11.56	16.19	25.96	29.79	36.16	45.15	39.39
12	18.59	2.35	8.40	9.45	21.52	40.74	45.34	46.58	48.38	59.24
13	10.38	2.12	7.00	9.61	9.04	27.51	26.96	32.23	47.50	63.41
14	125.41	1.20	2.66	3.72	15.18	24.54	35.98	42.48	59.02	46.01
15	6.81	1.18	2.85	6.68	5.44	7.94	12.16	9.02	11.36	16.99
16	9.01	1.47	3.42	6.89	8.28	34.56	23.06	28.84	37.77	65.64
17	7.22	1.12	3.28	3.77	6.54	18.36	17.80	26.83	26.85	32.13
18	Diver.	4.57	20.25	41.03	37.93	36.81	42.91	48.53	56.93	60.74
19	5.14	1.47	2.79	3.69	6.16	11.03	10.07	9.82	20.03	22.93
20	13.91	2.00	4.27	11.29	14.40	40.24	35.36	84.42	46.32	71.77
AveRecord (mm)	19.90	3.27	6.96	10.76	14.98	25.59	27.47	34.07	37.64	46.83
AveRecordDispSpect(R=3)	4.64	2.61	2.75	3.94	3.67	4.18	3.76	3.80	3.42	3.48

**Table 7:** DCR for longitudinal and hoop stresses under critical earthquake conditions for various storage tanks.

No.	H/R	Thick _min_ (mm)	$F_{allow}^{Long}$ (MPa)	$F_{allow}^{Hoop}$ (MPa)	DCR (Long)	DCR (Hoop)
1	0.3	6	20.68	183.81	26.60	1.45
2	0.6	6	29.52	183.81	9.49	1.14
3	0.9	6	32.44	183.81	12.08	1.45
4	1.2	6	34.90	183.81	14.04	1.52
5	1.5	6	37.06	183.81	11.33	1.37
6	1.8	6	39.02	183.81	3.59	0.84
7	2.1	8	46.34	183.81	4.32	0.91
8	2.4	10	53.53	183.81	5.60	1.45
9	2.7	12	60.62	183.81	6.47	1.22
10	3.0	14	67.63	183.81	4.97	1.29

**Table 8:** DCR of Longitudinal stress and hoop stress for critical earthquake in different tanks and optimal thickness.

No.	H/R	Thick_Opt_ (mm)	$F_{allow}^{Long}$ (MPa)	$F_{allow}^{Hoop}$ (MPa)	DCR (Long)	DCR (Hoop)
1	0.3	30	54.34	183.81	0.92	0.53
2	0.6	12	46.07	183.81	0.98	0.38
3	0.9	20	71.05	183.81	0.99	0.69
4	1.2	20	73.51	183.81	0.88	0.69
5	1.5	20	75.67	183.81	0.83	0.50
6	1.8	30	103.42	183.81	1.00	0.53
7	2.1	35	103.42	183.81	0.80	0.30
8	2.4	48	103.42	183.81	0.74	0.18
9	2.7	71	103.42	183.81	0.81	0.14
10	3	110	103.42	183.81	0.88	0.11

**Table 9:** Maximum displacement obtained from NLTHA for tanks designed according to API 650.

EQ	Maximum Displacement for H/R (mm)									
	0.3	0.6	0.9	1.2	1.5	1.8	2.1	2.4	2.7	3.0
1	0.83	0.57	0.56	0.90	1.19	1.34	2.29	2.54	4.38	3.02
2	1.91	2.53	2.85	2.32	6.62	5.33	7.22	5.95	20.46	5.00
3	2.72	1.51	1.52	2.94	3.11	3.30	2.64	3.74	13.85	4.34
4	5.02	1.30	1.74	5.16	9.22	28.06	11.91	11.62	32.99	17.59
5	2.05	1.47	1.72	2.15	3.69	3.96	5.60	9.70	11.18	10.26
6	1.81	0.82	0.68	2.04	3.88	3.81	5.31	4.32	6.66	5.83
7	3.17	2.23	2.70	3.55	5.44	6.22	4.74	9.80	13.24	10.82
8	7.50	2.57	5.30	8.33	6.00	8.58	6.77	9.43	17.88	13.26
9	3.00	1.57	1.67	2.73	4.20	3.30	4.52	4.24	11.41	4.56
10	0.95	0.67	0.70	1.06	1.51	1.55	2.75	3.09	6.77	3.54
11	1.50	0.84	1.04	1.46	4.73	4.34	3.82	4.32	14.75	5.04
12	1.77	1.80	2.26	1.89	2.54	1.79	3.01	5.73	10.61	7.08
13	1.88	0.83	0.92	2.02	2.38	2.41	2.86	4.92	12.27	5.53
14	0.74	0.55	0.53	0.79	1.05	1.13	1.64	2.39	3.24	2.34
15	0.81	0.57	0.58	1.12	1.24	1.05	1.09	1.35	4.17	2.05
16	0.70	0.56	0.56	0.94	1.14	1.39	1.97	2.33	5.59	2.79
17	0.73	0.47	0.53	0.81	1.29	1.26	1.94	3.18	4.43	3.56
18	2.23	1.23	2.14	2.40	8.07	4.86	5.90	7.85	29.62	9.28
19	0.85	0.55	0.52	0.92	1.41	1.24	1.81	2.31	3.27	2.68
20	1.50	1.40	1.13	1.38	1.67	1.74	2.87	3.02	4.22	4.50
AveRecord (mm)	2.08	1.20	1.48	2.24	3.52	4.33	4.03	5.09	11.55	6.15
AveRecordDispSpect(R=3)	0.49	0.96	0.59	0.82	0.86	0.71	0.55	0.57	1.05	0.46

## Data Availability

The original contributions presented in this study are included in the article. Further inquiries can be directed to the corresponding author.

## References

[1] Merino Vela R., Brunesi E., Nascimbene R. (2020). Probabilistic evaluation of earthquake-induced sloshing wave height in above-ground liquid storage tanks. Eng. Struct..

[2] Calvi G.M., Nascimbene R. (2023). Seismic Design and Analysis of Tanks.

[3] Jia J. (2016). Modern Earthquake Engineering: Offshore and Land-Based Structures.

[4] Rawat A., Mittal V., Chakraborty T., Matsagar V. (2019). Earthquake induced sloshing and hydrodynamic pressures in rigid liquid storage tanks analyzed by coupled acoustic-structural and Euler-Lagrange methods. Thin-Walled Struct..

[5] Eshghi S., Razzaghi M.S. (2007). Performance of cylindrical liquid storage tanks in Silakhor, Iran earthquake of March 31, 2006. Bull. N. Z. Soc. Earthq. Eng..

[6] Brunesi E., Nascimbene R., Pagani M., Beilic D. (2015). Seismic performance of storage steel tanks during the May 2012 Emilia, Italy, Earthquakes. J. Perform. Constr. Facil..

[7] Yazdanian M., Ingham J.M., Kahanek C., Dizhur D. (2020). Damage to flat-based wine storage tanks in the 2013 and 2016 New Zealand earthquakes. J. Constr. Steel Res..

[8] Zama S., Nishi H., Hatayama K., Yamada M., Yoshihara H., Ogawa Y. On damage of oil storage tanks due to the 2011 off the Pacific Coast of Tohoku Earthquake (Mw9. 0), Japan. Proceedings of the 15th World Conference on Earthquake Engineering (15WCEE).

[9] Fischer E.C., Liu J., Varma A.H. (2016). Investigation of cylindrical steel tank damage at wineries during earthquakes: Lessons learned and mitigation opportunities. Pract. Period. Struct. Des. Constr..

[10] Fischer E. Learning from Earthquakes: 2014 Napa Valley Earthquake Reconnaissance Report. https://docs.lib.purdue.edu/civlgradreports/1/.

[11] Korkmaz K.A., Sari A., Carhoglu A.I. (2011). Seismic risk assessment of storage tanks in Turkish industrial facilities. J. Loss Prev. Process Ind..

[12] Tsipianitis A., Tsompanakis Y. (2019). Impact of damping modeling on the seismic response of base-isolated liquid storage tanks. Soil Dyn. Earthq. Eng..

[13] Brunesi E., Nascimbene R. (2024). Evaluating the Seismic Resilience of Above-Ground Liquid Storage Tanks. Buildings.

[14] Housner G.W. (1963). The dynamic behavior of water tanks. Bull. Seismol. Soc. Am..

[15] Veletsos A.S., Tang Y., Tang H. (1992). Dynamic response of flexibly supported liquid-storage tanks. J. Struct. Eng..

[16] Malhotra P.K. (1997). Seismic response of soil-supported unanchored liquid-storage tanks. J. Struct. Eng..

[17] Bakalis K., Vamvatsikos D., Fragiadakis M. (2017). Seismic risk assessment of liquid storage tanks via a nonlinear surrogate model. Earthq. Eng. Struct. Dyn..

[18] Wu G., Ma Q., Taylor R. (1998). Numerical simulation of sloshing waves in a 3D tank based on a finite element method. Appl. Ocean. Res..

[19] Xue M.A., Chen Y., Zheng J., Qian L., Yuan X. (2019). Fluid dynamics analysis of sloshing pressure distribution in storage vessels of different shapes. Ocean Eng..

[20] Felix-Gonzalez I., Sanchez-Mondragon J., Cruces-Giron A. (2022). Sloshing study on prismatic LNG tank for the vertical location of the rotational center. Comput. Part. Mech..

[21] Merino R.J., Brunesi E., Nascimbene R. (2018). Derivation of floor acceleration spectra for an industrial liquid tank supporting structure with braced frame systems. Eng. Struct..

[22] Nascimbene R., Rassati G.A. (2024). Seismic Design and Evaluation of Elevated Steel Tanks Supported by Concentric Braced Frames. CivilEng.

[23] Nascimbene R., Fagà E., Moratti M. (2024). Seismic Strengthening of Elevated Reinforced Concrete Tanks: Analytical Framework and Validation Techniques. Buildings.

[24] Haroun M.A., Tayel M.A. (1985). Response of tanks to vertical seismic excitations. Earthq. Eng. Struct. Dyn..

[25] Park J.-H., Koh H., Kim J. (1992). Fluid-structure interaction analysis by a coupled boundary element-finite element method in time domain. Boundary Element Technology VII.

[26] Chen W., Haroun M.A., Liu F. (1996). Large amplitude liquid sloshing in seismically excited tanks. Earthq. Eng. Struct. Dyn..

[27] Hoskins L.M., Jacobsen L.S. (1934). Water pressure in a tank caused by a simulated earthquake. Bull. Seismol. Soc. Am..

[28] Kianoush M., Chen J. (2006). Effect of vertical acceleration on response of concrete rectangular liquid storage tanks. Eng. Struct..

[29] Veletsos A. Seismic effects in flexible liquid storage tanks. Proceedings of the 5th World Conference on Earthquake Engineering (5WCEE).

[30] Wu G., Eatock Taylor R., Greaves D. (2001). The effect of viscosity on the transient free-surface waves in a two-dimensional tank. J. Eng. Math..

[31] Estekanchi H., Alembagheri M. (2012). Seismic analysis of steel liquid storage tanks by endurance time method. Thin-Walled Struct..

[32] Constantin L., De Courcy J., Titurus B., Rendall T., Cooper J.E. (2021). Analysis of damping from vertical sloshing in a SDOF system. Mech. Syst. Signal Process..

[33] Jamshidi S., Firouz-Abadi R., Amirzadegan S. (2022). New mathematical model to analysis fluid sloshing in 3D tanks with slotted middle baffle. Ocean Eng..

[34] Huang S., Duan W., Han X., Nicoll R., You Y., Sheng S. (2018). Nonlinear analysis of sloshing and floating body coupled motion in the time-domain. Ocean Eng..

[35] Monaghan J.J. (1994). Simulating free surface flows with SPH. J. Comput. Phys..

[36] Shao J., Li H., Liu G., Liu M. (2012). An improved SPH method for modeling liquid sloshing dynamics. Comput. Struct..

[37] Gabbianelli G., Milanesi R., Gandelli E., Dubini P., Nascimbene R. (2023). Seismic vulnerability assessment of steel storage tanks protected through sliding isolators. Earthq. Eng. Struct. Dyn..

[38] Bagheri S., Farajian M. (2018). The effects of input earthquake characteristics on the nonlinear dynamic behavior of FPS isolated liquid storage tanks. J. Vib. Control.

[39] (2021). Welded Tanks for Oil Storage.

[40] (2016). Minimum Design Loads and Associated Criteria for Buildings and Other Structures.

[41] Veletsos A., Shivakumar P. (1997). Tanks containing liquids or solids. A Handbook in Computer Analysis and Design of Earthquake Resistant Structures.

[42] Buratti N., Tavano M. (2014). Dynamic buckling and seismic fragility of anchored steel tanks by the added mass method. Earthq. Eng. Struct. Dyn..

[43] Nascimbene R. (2024). Investigation of seismic damage to existing buildings by using remotely observed images. Eng. Fail. Anal..

[44] Mohammad K., Ruggieri S., Tateo V., Butenweg C., Nascimbene R., Uva G. (2025). Assessment of the seismic overpressure in flat bottom steel silos based on advanced FE modelling approach. Soil Dyn. Earthq. Eng..

[45] Quinci G., Paolacci F., Fragiadakis M., Bursi O. (2025). A machine learning framework for seismic risk assessment of industrial equipment. Reliab. Eng. Syst. Saf..

[46] Butenweg C., Bursi O., Paolacci F., Marinkovic M., Lanese I., Nardin C., Quinci G. (2021). Seismic performance of an industrial multi-storey frame structure with process equipment subjected to shake table testing. Eng. Struct..

